# Unusual A(H1N7) influenza A virus isolated from free-range domestic ducks in Bangladesh, 2023

**DOI:** 10.1128/mra.00218-24

**Published:** 2024-07-24

**Authors:** Jasmine C. M. Turner, David Walker, Md. Kamrul Hasan, Sharmin Akhtar, Subrata Barman, Nabanita Mukherjee, Pamela McKenzie, Richard J. Webby, Mohammed M. Feeroz

**Affiliations:** 1 Deptartment of Host-Microbes Interactions, St. Jude Children's Research Hospital, Memphis, Tennessee, USA; 2 Department of Zoology, Wildlife Rescue Center, Jahangirnagar University, Savar, Bangladesh; Portland State University, Portland, Oregon, USA

**Keywords:** surveillance, H1N7, unusual, ducks, Bangladesh, influenza, avian viruses

## Abstract

In Bangladesh, free-range duck farms provide opportunities for the generation of novel influenza A viruses as evidenced by the emergence of an unusual A(H1N7) virus in 2023. Continued surveillance of such environments for the potential emergence of influenza A viruses with novel properties remains a priority.

## ANNOUNCEMENT

Migratory waterfowl serves as the primary reservoir for influenza A viruses (IAVs) (1).Longitudinal surveillance studies on influenza A circulation were conducted from January 2022 to March 2023 in a wetland region of Bangladesh, Berberia Beel, Tanguar Haor, where free-range domestic ducks have frequent contact with migratory birds. Oropharyngeal and cloacal samples were collected from 179 Khaki Campbell ducks on a farm in Tanguar Haor. Swabs collected were placed in a phosphate buffered saline (PBS)/glycerol isolation medium and stored in liquid nitrogen. Upon arrival, samples were screened for influenza A via real-time reverse transcription PCR (RT-PCR) using primers designed by the Centers for Disease Control and Prevention according to established protocols (2, 3).

Eighty-eight influenza A positive samples were injected into 10-day-old embryonated chicken eggs. All eighteen isolates obtained were verified as influenza A viruses via Illumina sequencing, placing them in the family *Orthomyxoviridae*. Viral RNA was extracted from allantoic fluid by using Qiagen RNeasy mini kit (Cat. no. 74106) according to the manufacturer’s instructions. A two-step RT-PCR using Superscript IV First-Strand Synthesis System (Invitrogen, Cat. no. 18091050) and Phusion High-Fidelity DNA Polymerase (New England Biolabs, Cat. no M0530L) was performed to amplify all eight segments of the influenza genome according to published protocols (4, 5). Individual libraries were prepared for each isolate from 1 ng of amplicon with the Nextera XT DNA Library Preparation Kit (Illumina PN FC-131–1096). Libraries were quantified and analyzed for insert size distribution using the 2100 BioAnalyzer High Sensitivity kit (Agilent). Libraries were sequenced (paired-end 2 × 150) on a MiSeq (Illumina) using v2 flowcells and reagent kits as per the manufacturer’s protocol. Reads were demultiplexed, quality-trimmed, filtered, and *de novo* assembled, and contigs were analyzed using CLC Genomics Workbench version 21.0.1 and BioEdit Sequence Alignment Editor version 7.25 for alignment. Total reads for all 18 virus isolates ranged from 99,294 to 185,850, with an average length ranging from 183.54 to 201.62 base pairs and 44% GC content.

**Fig 1:**
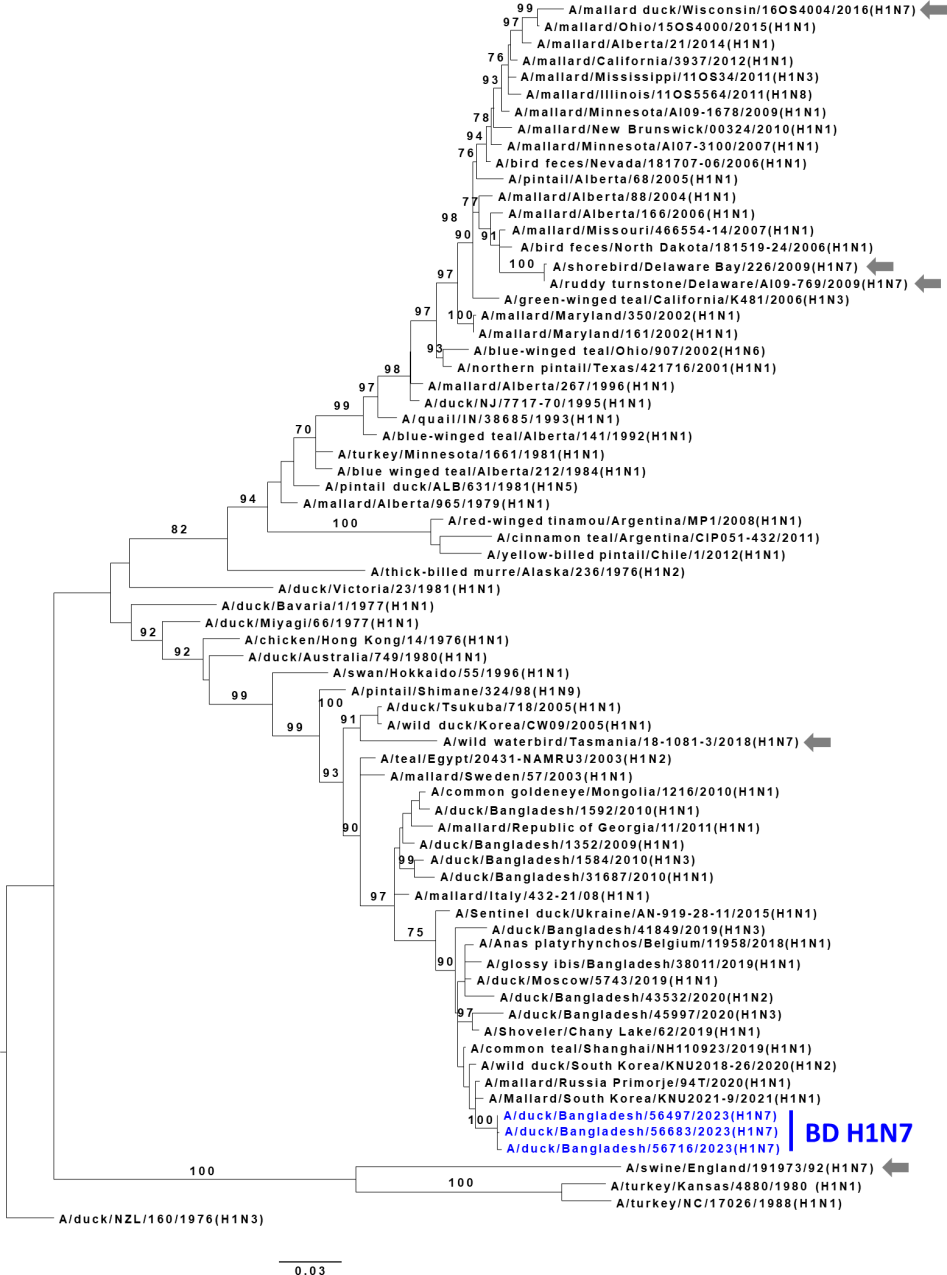
Phylogenetic relationship of hemagglutinin genes of H1N7 viruses isolated in Bangladeshi live poultry markets (LPMs). Viruses isolated and characterized during the current surveillance period are shown in color. Gray arrows show previously isolated H1N7 viruses. Trees are rooted to midpoint, and bootstrap values ≥70% are specified on the branches.

For phylogenetic analysis, sequences other than those in this study were retrieved from the EpiFlu database of the Global Initiative on Sharing All Influenza Data (6) and the National Center for Biotechnology Information Influenza Virus Sequence Database (7). Previously aligned sequences were trimmed to equal lengths. Phylogenetic relationships were inferred by using the Maximum Likelihood method and Tamura-Nei model (8). The reliability of phylogenetic inference at each branch node was estimated by the bootstrap method with 1,000 replications. Evolutionary analyses were conducted in MEGA 7 (9). All tools were run with default parameters unless otherwise specified (Fig. 1).

All 18 virus genomes were of the A(H1N7) subtype as determined by BLASTn searches of GenBank (10). All eight gene segments of the A(H1N7) viruses share >97% nucleotide identity with some viruses isolated from different migratory birds across Europe and Asia between 2015 and 2021 (Table 1). The amalgamation of these gene segments into a viable and rarely identified IAV subtype originating in domestic ducks at the wild bird-poultry-human interface highlights the importance of continued surveillance for the possible emergence of a novel influenza virus with pandemic potential.

**TABLE 1:** Nucleotide identity and genome analysis of novel LPAI A(H1N7) virus gene segments with other viruses

Gene^*a*^	Size (nucleotide)	Mapped reads^*b*^	Depth of coverage	Full genome GC content (%)	Most closely related virus strain	% Identity (nucleotide)
PB2	2,341	10,587	854.97	44.7	A/mallard/Dagestan/1050/2018(H7N3)	98
PB1	2,341	10,632	828.88	44.7	A/pheasant/Italy/21VIR2284-1/2021(H9N2)	98
PA	2,233	14,235	1,215.22	44.7	A/Chicken/Sweden/SVA210217SZ0001/KN001190-IP6/2021(H5N8)	98
HA	1,775	16,018	1,806.45	44.7	A/mallard/Russia Primorje/94T/2020(H1N1)	98
NP	1,565	17,991	2,288.08	44.7	A/mallard/Denmark/09179-5/2022 (H5N2)	98
NA	1,409	19,637	2,404.40	44.7	A/common teal/Chany Lake/4/2020(H10N7)	97
M	1,027	20,958	3,988.11	44.7	A/mallard/Ukraine/AN-221–13-01/2020 (A/H7N2)	98
NS	890	12,004	2,648.07	44.7	A/mallard/South Korea/34X-2/2021 (A/H7N7)	99

^*a*^ Nucleotide sequences from all eight gene segments of a representative H1N7 isolate, A/duck/Bangladesh/56497/2023, were used for comparative analyses due to the >99% similarity of all 18 isolates obtained in this study.

^*b*^ The total number of mapped reads is 122,296 out of 123,624 total reads.

## Data Availability

The influenza A viral genome sequences for H1N7 subtype have been deposited in GenBank under the following accession numbers:A/duck/Bangladesh/56577/2023(H1N7) OR664416-OR664423, A/duck/Bangladesh/56485/2023(H1N7) OR664639-OR664646, A/duck/Bangladesh/56497/2023(H1N7) OR664432-OR664439, A/duck/Bangladesh/56523/2023(H1N7) OR664424-OR664431, A/duck/Bangladesh/56564/2023(H1N7) OR664539-OR664546, A/duck/Bangladesh/56589/2023(H1N7) OR664562-OR664569, A/duck/Bangladesh/56659/2023(H1N7) OR664408-OR664415, A/duck/Bangladesh/56665/2023(H1N7) OR664531-OR664538, A/duck/Bangladesh/56681/2023(H1N7) OR664507-OR664514, A/duck/Bangladesh/56683/2023(H1N7) OR664448-OR664455, A/duck/Bangladesh/56707/2023(H1N7) OR664585-OR664592, A/duck/Bangladesh/56711/2023(H1N7) OR664648-OR664654, A/duck/Bangladesh/56716/2023(H1N7) OR664616-OR664623, A/duck/Bangladesh/56727/2023(H1N7) OR664601-OR664608, A/duck/Bangladesh/56731/2023(H1N7) OR664478-OR664484, A/duck/Bangladesh/56746/2023(H1N7) OR664554-OR664561, A/duck/Bangladesh/56755/2023(H1N7) OR664485-OR664492, and A/duck/Bangladesh/56791/2023(H1N7) OR664524-OR664530. All raw reads were submitted to NCBI SRA under the following accession numbers: BioProject PRJNA298519, BioSamples SAMN3396029 - SAMN3396046
